# Case Report: Patent ductus arteriosus with tuberous sclerosis complex

**DOI:** 10.3389/fcvm.2024.1391775

**Published:** 2024-07-25

**Authors:** Tingrui Chen, Xiaoxiao Wu, Yiping Wang

**Affiliations:** Department of ICU, Sichuan Provincial People’s Hospital, University of Electronic Science and Technology of China, Chengdu, China

**Keywords:** patent ductus arteriosus, tuberous sclerosis complex, gene, ICU, TSC

## Abstract

A 33-year-old patient presented with a chief complaint of patent ductus arteriosus (PDA) persisting for over 30 years. Physical examination revealed bilateral facial angiofibromas, multiple nail fibromas, intraoral fibromas, and a ’shagreen patch’ on the left lumbar region. Genetic testing was performed using a peripheral venous blood sample, which confirmed the diagnosis of Tuberous Sclerosis Type 2 (TSC2). Subsequently, the patient underwent cardiac color Doppler ultrasound and chest computed tomography angiography, which confirmed the presence of PDA. Tuberous sclerosis complex (TSC) is associated with cardiovascular diseases. The initial clinical manifestation of TSC is usually cardiac rhabdomyoma in children, and it is rarely reported in adults with PDA. In this case, the patient was diagnosed with PDA when he was young, and the genetic test showed heterozygous variation of TSC2 gene. The purpose of this article is to explore the correlation between TSC and PDA at the gene level through literature review.

## Introduction

Tuberous sclerosis complex (TSC) is characterized by the presence of multiple tumors in organs such as the brain, heart, skin, and kidneys. It affects approximately 1 in 6,000–10,000 individuals, classifying it as a rare disease. The term “TSC” was first coined by Sylvan Moolten in 1942. Hypomelanotic macules and patches (also known as ash leaf spots) are common diagnostic criteria, as 90% of TSC patients exhibit this symptom. These lesions often manifest at birth or become apparent during infancy. As individuals age, the number of these macules may increase, and other skin features such as facial angiofibromas, fibrous cephalic plaques, and shagreen patches may become more prominent. TSC has demonstrated a highly variable clinical expressivity and complete penetrance. Genetic diagnosis serves as an independent diagnostic criterion for confirming TSC. Pathogenic mutations in the TSC1 and TSC2 genes, including insertions, deletions, mutations, and large fragment gene deficiency, as well as missense mutations known to affect protein function, can be used as diagnostic markers to establish a clear TSC diagnosis. However, a small portion of TSC patients may not have detectable TSC1 or TSC2 gene mutations. Therefore, a negative genetic test does not exclude the diagnosis of TSC, and these patients may be diagnosed based on clinical features alone.

## Case report

**Figure 1:**
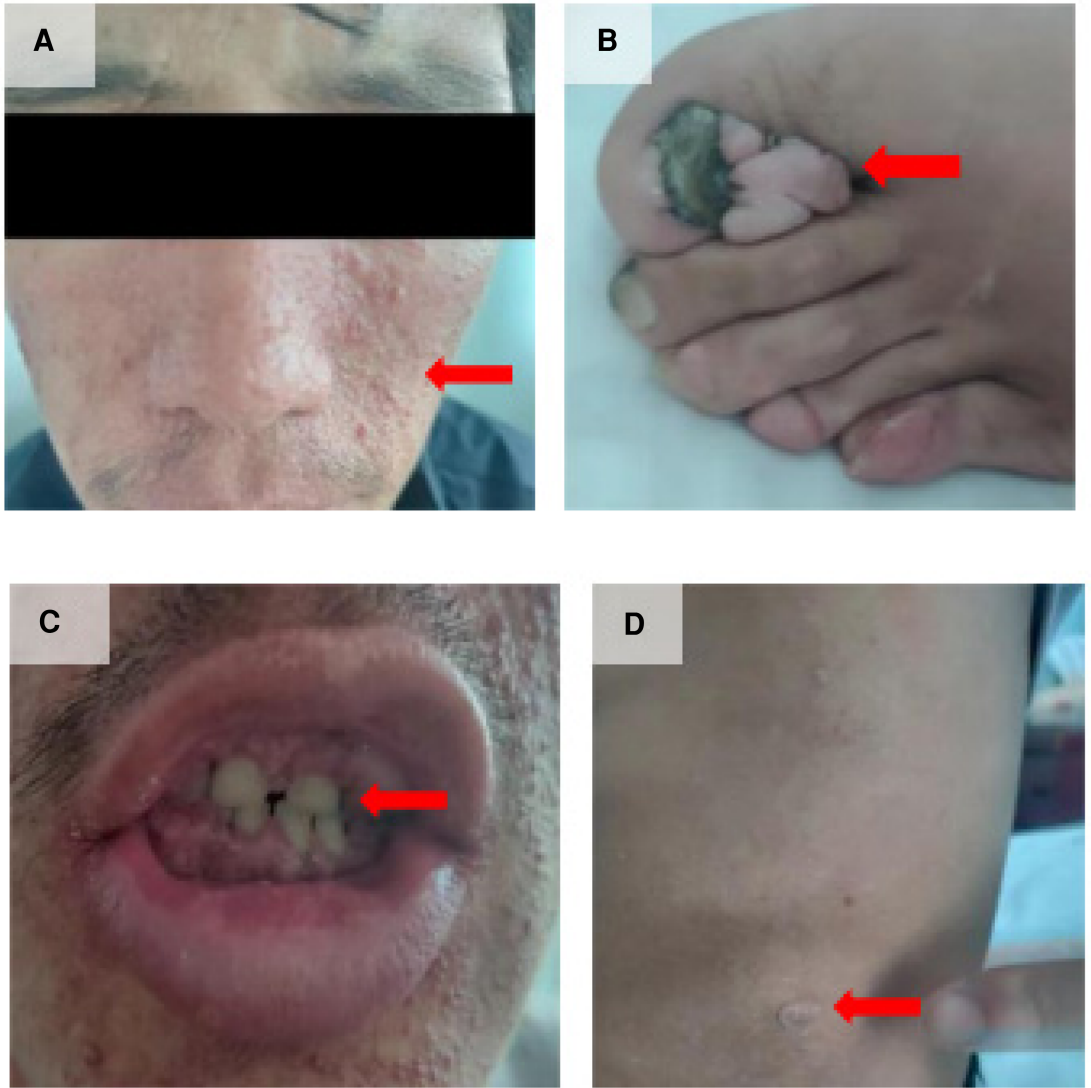
Clinical manifestations of the patient. (**A**) Facial angiofibromas; (**B**) Multiple toenail fibromas; (**C**) Fibromas in the oral cavity; (**D**) “Shark skin” lies on the left lower back.

A 33-year-old man with a history of patent ductus arteriosus (PDA) diagnosed over 30 years ago was admitted to our hospital six months ago. At the age of 3, the patient was diagnosed with PDA. Approximately 3 months ago, the patient experienced syncope with transient loss of consciousness without any obvious trigger. He sought medical attention at a local hospital, where a cardiac echocardiogram revealed congenital heart disease with PDA (bilateral shunting predominantly from right to left at the great vessel level), an echogenic septum in the descending aorta, moderate aortic valve stenosis, and mild aortic and mitral valve regurgitation. The patient has no history of smoking, alcohol consumption, or other harmful habits. The patient got married at 27 and did not have children. Both his parents are in good health, and his younger brother is physically healthy. There is no family history of hereditary or genetic diseases. Upon physical examination, the patient appeared to be of normal development. Decreased breath sounds were noted in both lungs, and no dry or wet rales were heard. Symmetrically distributed pinpoint to millet-sized red papules were observed on the patient's face, particularly centered around the nose (Figure 1A). The total area was approximately 5 cm × 8 cm on the left side and 5 cm × 3 cm on the right side. On the left foot, several tumor-like growths of varying sizes ranging from 2 to 17 mm in diameter were observed along the peritoenail. The tumors had smooth surfaces and were firm in texture (Figure 1B). There was mild cyanosis of the lips, significant gingival hyperplasia covering two-thirds of the dental crowns, increased spacing between teeth, and a slight malocclusion with fibrous papules present inside the oral cavity (Figure 1C). On the left side of the lumbar region, a papule measuring 2 cm × 2 cm was visible (Figure 1D).

## Investigations

**Figure 2:**
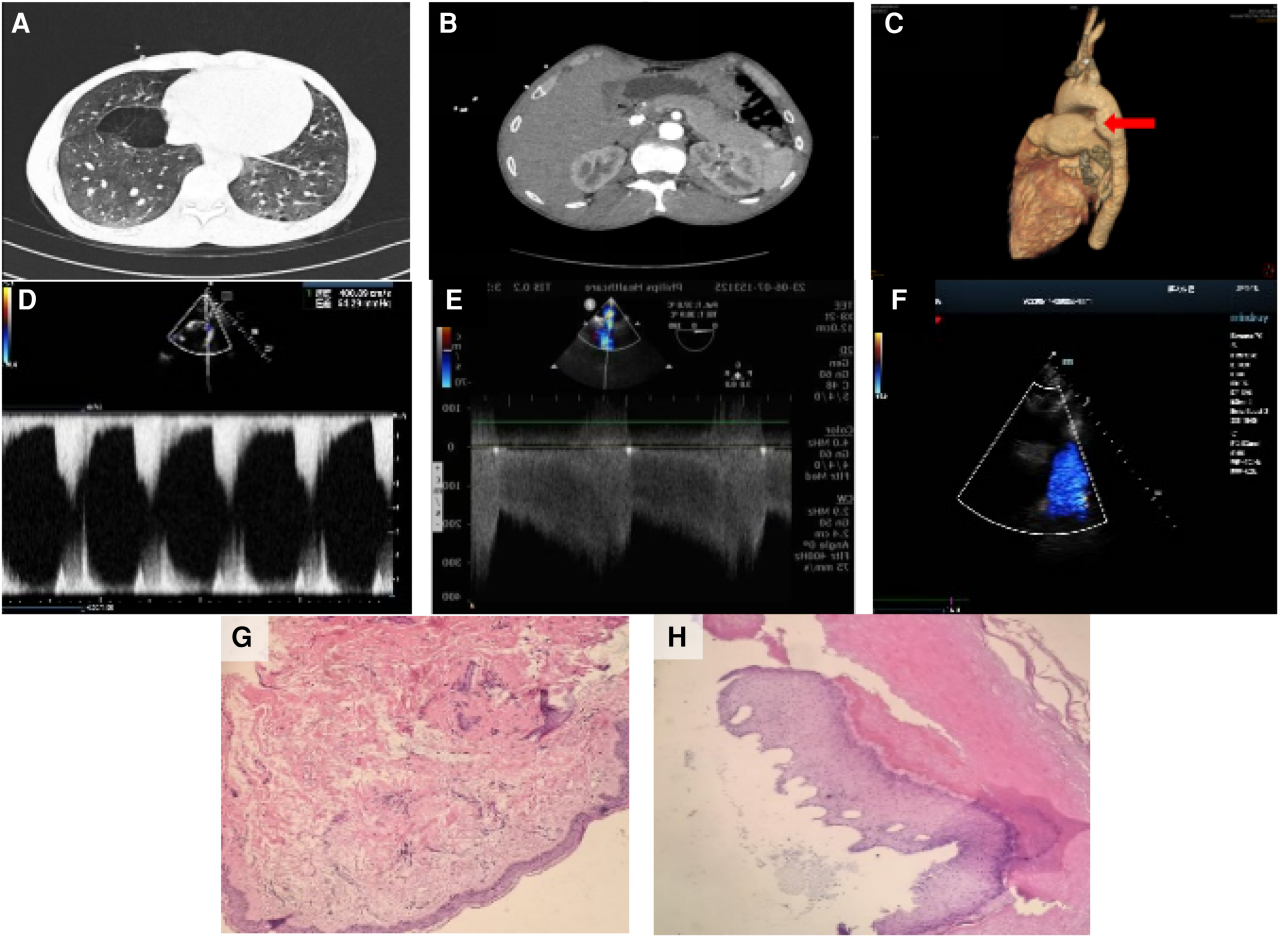
Imaging and pathology results. (**A**) Several solid nodules in both lungs, with the largest one located in the anterior segment of the right upper lobe, approximately 8 mm × 7 mm in size. Emphysema with pulmonary bullae was also observed in both lungs. Preoperative abdominal plain CT scan. (**B**) Nodular dense shadow in the liver, nodular dense shadow in the duodenal region, and several nodules in both kidneys, with the largest one having a long diameter of about 64 mm. Preoperative three-dimensional imaging of the thoracic aorta. (**C**) Fenestration patent ductus arteriosus with pulmonary hypertension. (**D**) Before the operation, anecho interruption was observed between the origin of the left pulmonary artery and the descending aorta, approximately 13 mm in width (window type), with a continuous left-to-right shunt detected at the level of the large artery. Vmax was approximately 3.2 m/s, and Vmin was approximately 1.7 m/s. (**E**) During the operation, the occluder-like echo was detected between the origin of the left pulmonary artery and the descending aorta. The position was fixed, the traction test was negative, and no significant blood flow acceleration was detected at the origin of the descending aorta and the origin of the left pulmonary artery. (**F**) After the operation, the thickness of the interventricular septum and left ventricular posterior wall was normal. The overall motion amplitude of the left ventricular wall was slightly decreased, the diameter of the ascending aorta was widened, and the occluder-like hyperecho was detected between the origin of the left pulmonary artery and the descending aorta. (**G**) Pathological examination of the skin on the left waist showed hyperkeratosis of the epidermis and a few lymphocytes infiltration around the small vessels of the dermis. (**H**) Pathological examination of the skin on the left foot also showed hyperkeratosis of the epidermis and a few lymphocytes infiltration around the small vessels of the dermis.

Preoperative chest CT scan: several solid nodules in both lungs, the large one was located in the anterior segment of the right upper lobe, about 8 mm × 7 mm in size, emphysema in both lungs with pulmonary bullae). Preoperative abdominal plain CT scan: nodular dense shadow in the liver, nodular dense shadow in the duodenal region, and several nodules in both kidneys, the large one with a long diameter of about 64 mm). Preoperative three-dimensional imaging of the thoracic aorta: fenestration PDA with pulmonary hypertension. Before operation, there was an echo interruption between the origin of the left pulmonary artery and the descending aorta, about 13 mm in width (window type), and a continuous left-to-right shunt could be detected at the level of the large artery, with Vmax of about 3.2 m/s and Vmin of about 1.7 m/s. During the operation, the occluder-like echo could be detected between the origin of the left pulmonary artery and the descending aorta, the position was fixed, the traction test was negative, and the exact blood flow acceleration was not detected at the origin of the descending aorta and the origin of the left pulmonary artery. Pathological examination of the skin on the left waist and left foot showed hyperkeratosis of the epidermis and a few lymphocytes infiltration around the small vessels of the dermis (Figure 2). With the consent of the patient and his family members, blood samples were collected for genetic testing. Targeted region capture-based high-throughput sequencing was employed to sequence approximately 20,000 functional exons encoding genes in the human genome, as well as the mitochondrial genome. A heterozygous variant of the TSC2 gene was detected in the proband, supporting the clinical diagnosis of TSC2 (Table 1).

**Table 1:** Results of genetic testing for inherited genetic disorders.

Genes	Chromosomal location	Transcript exon	Nucleotide amino acids	Homozygous/ heterozygous	Proper pair according to frequency	Prediction	Pathogenicity analysis	Genetic sytle	Diseadse/phenotype	Mutation sources
TSC2	Chr16:212747	NM_000548. 5:Intron25	c.2838-122G>A	Het	–	–	Pathopoiesia	AD	The tuberous sclerosis complex type 2	Unknown

## Treatment

The patient was admitted to the department of cardiac surgery six months ago. Oral treatment with Sildenafil (25 mg q8h) and Ambrisentan (5 mg qd) was initiated before surgery. On June 7, 2023, under general anesthesia and ultrasound guidance, a transcatheter closure of the PDA was performed through a small thoracic incision. Intraoperative transesophageal echocardiography revealed a PDA with a diameter of 13 mm, presenting as a window type defect with left-to-right shunting and pulmonary artery hypertension.

## Outcome and follow-up

Postoperative echocardiography showed that the thickness of interventricular septum and left ventricular posterior wall was normal, the overall motion amplitude of left ventricular wall was slightly decreased, the diameter of ascending aorta was widened, and the occluder-like hyperecho could be detected between the origin of left pulmonary artery and descending aorta. The patient was admitted to ICU and continued supportive treatment such as pulmonary artery lowering, anticoagulation, and fluid infusionOn the third day after surgery, the patient was transferred out of the ICU and returned to the cardiac surgery departement for further treatment. The patient was prescribed oral treatment with Ambrisentan (5 mg qd) and enteric-coated aspirin (200 mg qd). On June 15, 2023, the patient was discharged according to medical advice. No symptoms such as syncope or chest tightness were reported after discharge.

## Discussion

Tuberous sclerosis complex (TSC) is an autosomal dominant genetic disease characterized by the presence of hamartomas affecting multiple systems in the body, caused by loss-of-function mutations in the TSC1 or TSC2 genes. When TSC is associated with cardiovascular system diseases, cardiac rhabdomyomas are most commonly observed in children (Table 2) and are often the initial clinical manifestation of TSC (1). The coexistence of TSC and patent ductus arteriosus (PDA) is rare, and case reports have indicated a poor prognosis (2). The present patient was diagnosed with PDA at a young age and was not treated, which was related to poor family economic conditions. His genetic testing conducted during the current visit revealed a heterozygous variation in the TSC2 gene, confirming the diagnosis of TSC.

**Table 2:** Similar cases reported in recent years.

Reference	Study type	Year of publication	Disease	Age of patients	Therapeutic regime	Outcomes
Obeidat M, et al.	Case report	2018	Tuberous sclerosis with Rhabdomyoma	Newborn	Blalock-Taussig shunt	Hospital discharge
Jiang ZY, et al.	Case report	2000	Tuberous sclerosis with Multiple Cardiac Rhabdomyomas	Newborn	–	Died at 2.5 months of age
Mercan I, et al.	Case report	2020	Rhabdomyoma with atri-oventricular septal defect	3 months old	Atrioventricular septal defect repair	PDA was completely closed
Ilina MV, et al.	Case report	2007	Rhabdomyoma Causing Right Ventricular Inflow Obstruction	Newborn	ductal stenting	Not mentioned

**Figure 3:**
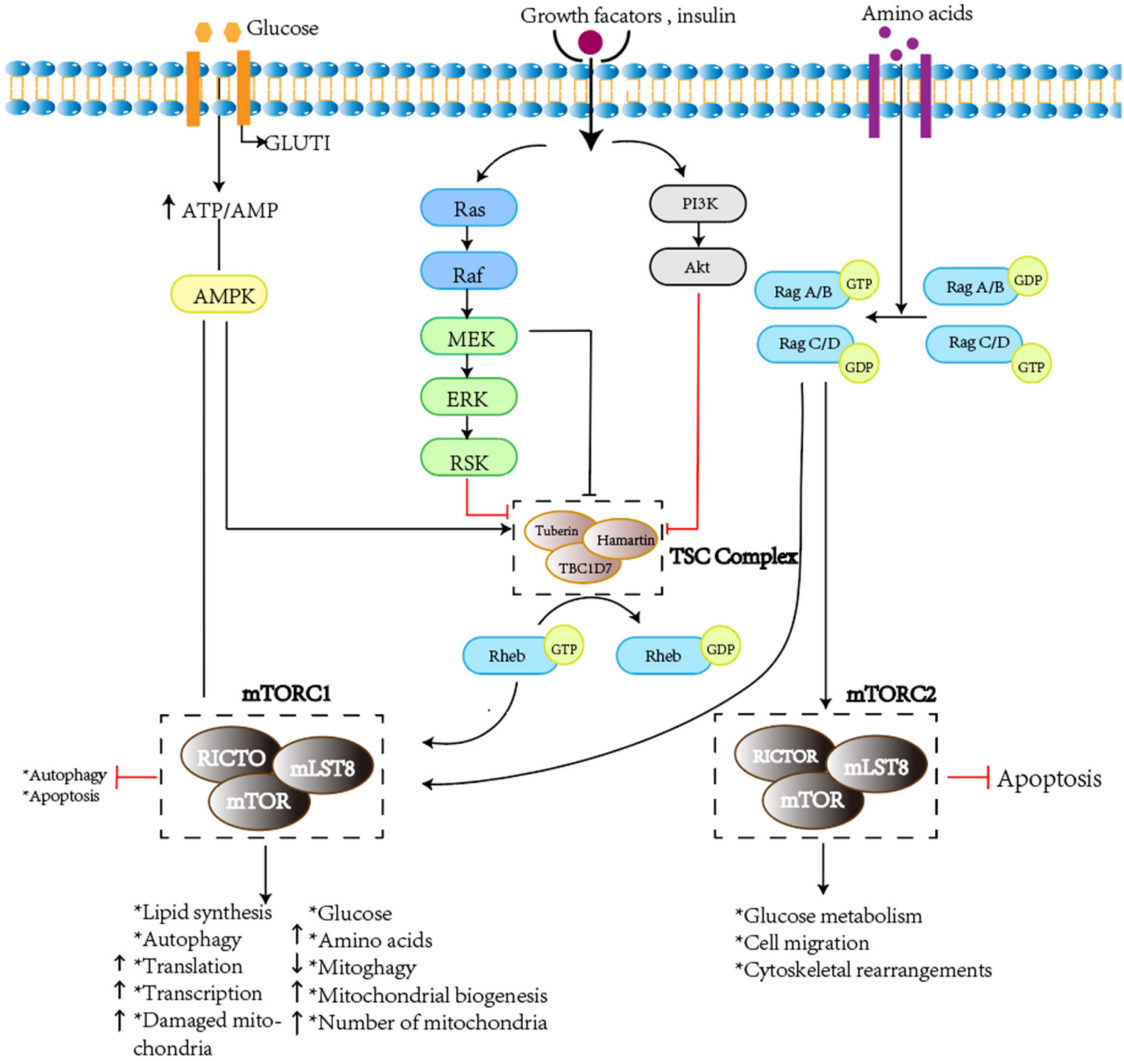
Pathogenic mechanisms of tuberous sclenosis complex (authors' original work). Dysfunction of the TSC complex causes mTORC1 hyperactivation through Rheb, which leads to metabolic and molecular changes. The TSC complex deactivates the RAS homolog enriched in brain (Rheb) by causing GTP to be cleaved from it. After stimulation by growth factor, the TSC complex is phosphorylated and its GTPase-activating protein activity is decreased. Similarly, dysfunction of the TSC complex is caused by loss of unction mutations of TSC1/TSC2 in the tuberous sclerosis complex (TSC). All of these factors activate Rheb to stimulate mammalian target of rapamycin (mTOR) complex 1 (mTORC1). mTORC1 directly regulates lipid, nucleotide, and protein synthesis to promote cell cycle progression and also inhibits autophagy. It ultimately causes excessive division and proliferation of cells to form hamartomas in multiple organs. AKT RACα serine/threonine-protein kinase; ERK extracellular-signal-regulated kinase; GLUT1 solute carrier family 2, facilitated glucose transporter member 1 (also known as glucose transporter type 1,erythrocyte/brain); mLST8 target of rapamycin complex subunit LST8; mTORC2 mammalian target of rapamycin complex 2; Raptor regulatory-associated protein of mTOR; RICTO rapamycin insensitive companion of mTOR; ROS reactive oxygen species; SLC1A5 neutral amino acid transporter B(0); TBC1D7 TBC1 domain family member 7.

The association between TSC and PDA remains unclear. TSC is caused by loss-of-function mutations in the TSC1 or TSC2 genes, which act as tumor suppressor genes. Inactivation of TSC1 or TSC2 leads to an increase in GTP-bound Rheb, a modulator of mechanistic target of rapamycin (mTOR) activity. The mTOR signaling pathway regulates cell growth and differentiation (3). TSC1 or TSC2 mutations result in the inactivation of TSC protein complexes, leading to the loss of inhibitory effects on the mTOR pathway (4) (Figure 3). However, studies have shown a significant increase in the expression of PTGS2 and prostacyclin synthase (PGTIS) in TSC2-deficient cells. On the other hand, elevated expression of EP3 (prostaglandin E3 homologous receptor) has been observed in cells derived from patients with TSC2 defects (5). Research has indicated that circulating levels of prostacyclin metabolites have been correlated with the severity and treatment response of PDA. The prostaglandin pathway plays a critical role in both functional and anatomical closure of the ductus arteriosus and is considered a valuable pharmacological target in maintaining ductal patency (6). COX-1 and COX-2 are prostaglandin synthetases, and COX-2 is a rate limiting enzyme that catalyzes the conversion of arachidonic acid to prostaglandins. Its overexpression has been documented in human tumors, and prostaglandins play a certain role in cancer development (7). Prostaglandin E2 (PGE2) is considered a major mediator of ductal relaxation, primarily acting through the EP4 receptor and inducing ductal dilation via activation of the cAMP/PKA pathway8. Therefore, we speculate that the absence of TSC2 may lead to an increased production of prostacyclin, thereby increasing the probability of PDA occurrence (8). The patient in this case was discharged after undergoing closure of the PDA without specific treatment for TSC.

## Data Availability

The original contributions presented in the study are included in the article/Supplementary Materials, further inquiries can be directed to the corresponding author.
